# Supplementary material for: Divergent evolution of sleep in Drosophila species
Source: Nat Commun. 2024 Jun 14;15:5091. doi: 10.1038/s41467-024-49501-9 (PMC11178934; doi:10.1038/s41467-024-49501-9)
Supplement: Supplementary file 1 — Supplementary Information [file 41467_2024_49501_MOESM1_ESM.pdf]

Divergent evolution of sleep in Drosophila species
Supplementary Material

Michaela Joyce, Federica A. Falconio, Laurence Blackhurst, Lucia Prieto-Godino , Alice S. French, and Giorgio F. Gilestro <*giorgio@gilest.ro*>

Transition probability matrix for *D.melanogaster*

	Deep sleep	Light sleep	Light awake	Active awake
Deep sleep	0.834628	0.0101301	0.155242	0
Light sleep	0.112523	0.672489	0.214988	0
Light awake	0	0.25632	0.731348	0.0123321
Active awake	0	0	0.0206226	0.979377

Transition probability matrix for *D.simulans*

	Deep sleep	Light sleep	Light awake	Active awake
Deep sleep	0.8411	0.117954	0.0409463	0
Light sleep	0.148504	0.630581	0.220914	0
Light awake	0	0.464499	0.411513	0.123988
Active awake	0	0	0.0942601	0.90574

Transition probability matrix for *D.sechellia*

	Deep sleep	Light sleep	Light awake	Active awake
Deep sleep	0.77786	0.205783	0.0163568	0
Light sleep	0.124332	0.63213	0.243538	0
Light awake	0	0.311411	0.675898	0.0126907
Active awake	0	0	0.0151483	0.984852

Transition probability matrix for *D.erecta*

	Deep sleep	Light sleep	Light awake	Active awake
Deep sleep	0.907331	0.089296	0.00337265	0
Light sleep	0.0307962	0.73296	0.236244	0
Light awake	0	0.4502	0.490159	0.0596405
Active awake	0	0	0.0793572	0.920643

Transition probability matrix for *D.yakuba*

	Deep sleep	Light sleep	Light awake	Active awake
Deep sleep	0.929505	0.0495132	0.0209817	0
Light sleep	0.103608	0.623525	0.272867	0
Light awake	0	0.368421	0.572346	0.0592332
Active awake	0	0	0.0292231	0.970777

Transition probability matrix for *D.willistoni*

	Deep sleep	Light sleep	Light awake	Active awake
Deep sleep	0.949612	0.0403958	0.00999263	0
Light sleep	0.047418	0.721462	0.23112	0
Light awake	0	0.327386	0.615529	0.0570855
Active awake	0	0	0.0570763	0.942924

Transition probability matrix for *D.virilis*

	Deep sleep	Light sleep	Light awake	Active awake
Deep sleep	0.790638	0.191275	0.0180869	0
Light sleep	0.148625	0.659888	0.191487	0
Light awake	0	0.358497	0.600394	0.0411089
Active awake	0	0	0.0553023	0.944698

Supplementary Table 1 | Sleep stages transition probability matrices for all species.
The table show all the likelihoods of transition between stage by species as calculated by the HMC model. Values are between 0 (never) and 1 (always). The pairing should be read vertically.

Figure	*Panel*	Authors
Fig. 1	c	MJ, ASF
	d,e	MJ
	f-h	MJ, LB
Supplementary Fig. 1	a-c	MJ
	d,e	MJ, LB
Supplementary Fig. 2	a,b	LB, GFG
Fig. 2	a,b	MJ
	c	MJ, LB
	d	MJ, FAF
Supplementary Fig. 3	a	ASF
	b-d	MJ
Supplementary Fig. 4, 5, 6	a	MJ, FAF
Supplementary Fig. 7	b	MJ, ASF
Fig. 3	a	MJ, GFG
	b-c	MJ
Supplementary Fig. 8	a-b	MJ
Fig. 4	a-b	MJ, ASF

Supplementary Table 2 | Detailed author contributions to experimental collection and data analysis.
Detailed authors'contribution to the experimental work in each panel, for main and supplementary figures. Authors are indicated according to their names' initials. All authors contributed to the writing and editing of the manuscript.

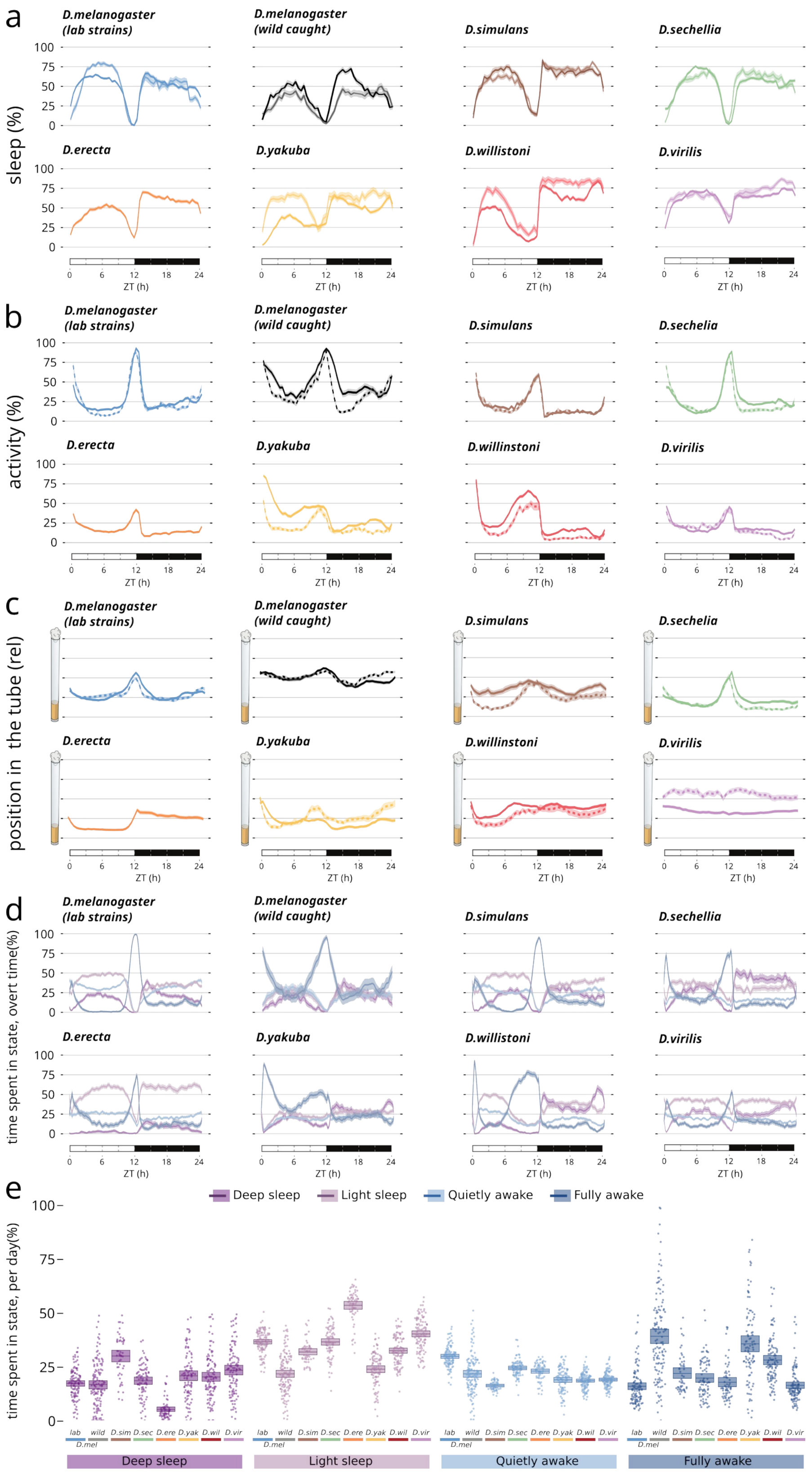

Supplementary Figure 1 | Characteristics of baseline sleep in the tested species.
a, Quantification of sleep amount over 24 h in all tested species. Except for *D. erecta*, two different geographically independent strains were analysed for all species, indicated by different shades of colour. The two *D. melanogaster* laboratory strains analysed are CantonS and OregonR. **b,** Activity profile and **c,** average position inside the experimental tube over 24 h for the strains shown in a. **d,** Sleep stages in all seven species during the 24 h period. **e,** Fraction of time spent in each given state over the 24 h period in all tested species.

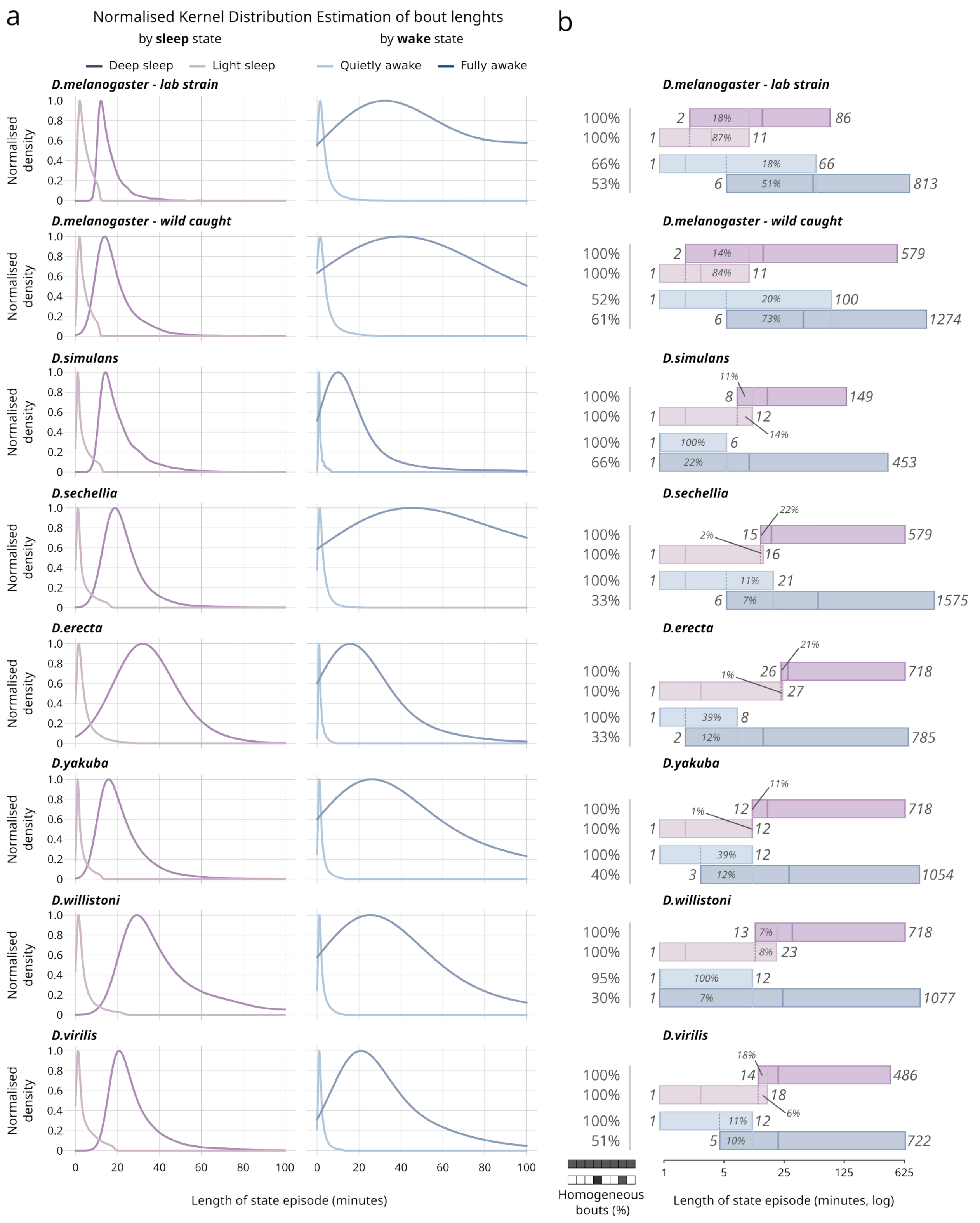

Supplementary Figure 2 | Defining characteristics of HMM-defined behavioural bouts.
a, Normalised Kernel density estimation (KDE) of the distribution of behavioural bouts by length in minutes on all species analysed. **b,** Quantitative description of bouts by type. The numbers on each end of the boxplots indicate the minimum (left) and maximum (right) values in minutes for each bout type. The vertical bar in each box indicates the median value for that bout. The percentages inside each box indicate the fraction of bouts of that state that have lengths overlapping with the adjacent state and are therefore disambiguated by the Hidden Markov Chain model based only on transition probability and not bout length. Percentages on the left indicate the proportion of homogenous bouts, that is bouts that are composed of only inactivity for sleep or only activity for waking (only for bouts longer than 2 minutes).

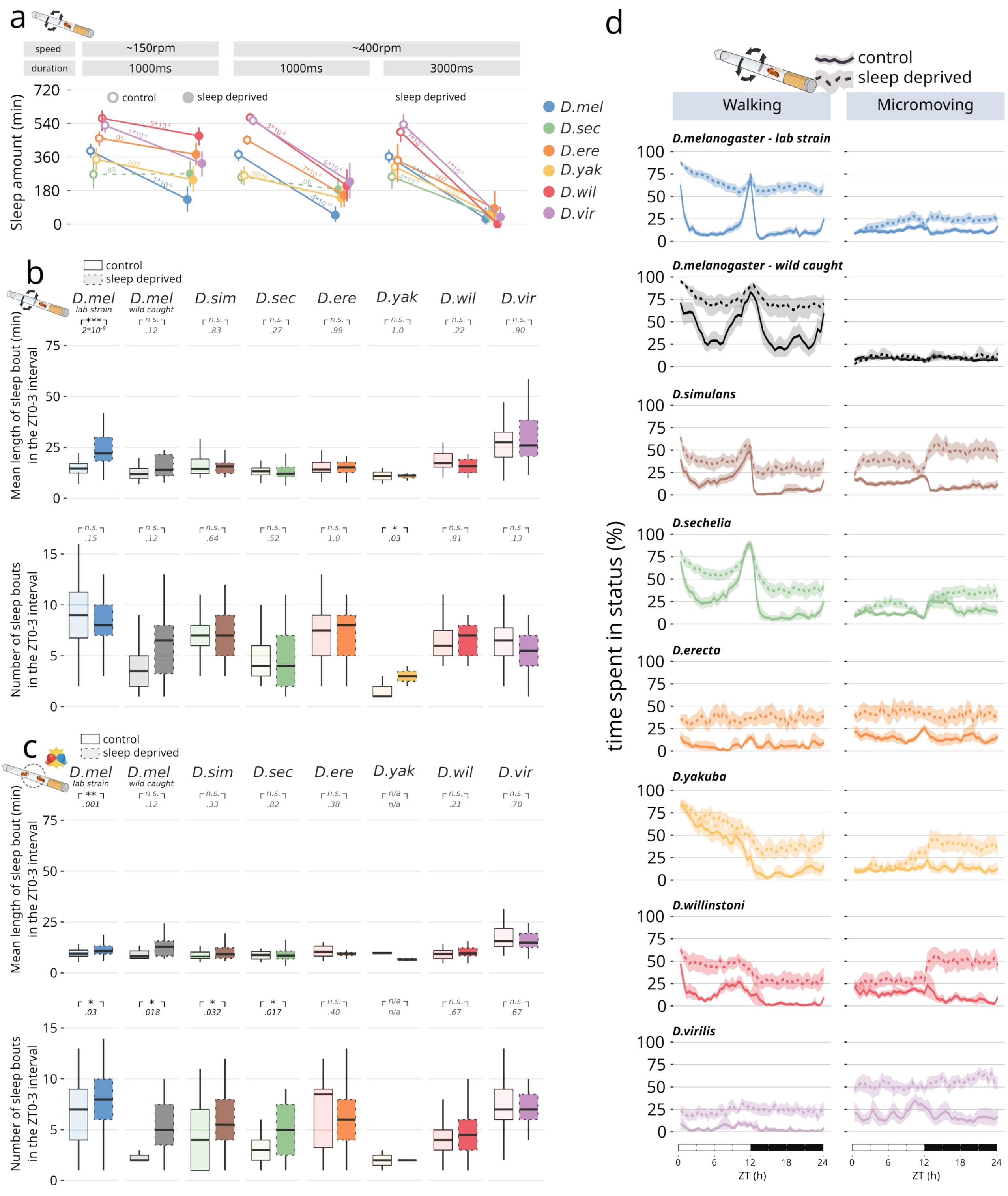

Supplementary Figure 3 | Characteristics of sleep deprivation and rebound in the tested species.
a, Quantification of sleep in six *Drosophila* species during the sleep deprivation night using three different settings of mechanical stimuli varying speed (~150-400 revolutions per minute) and duration (1-3 seconds). P-values are shown for each comparison. Dashed lines indicate P-values above 0.05. **b,** Mean length of sleep bouts and mean number of sleep bouts at ZT 0-3 in control (continuous lines) or animals mechanically sleep-deprived (dashed). **c,** same as b but with male-male sleep deprivation. **d,** Quantification of behaviour across the 24 h in mechanically sleep-deprived animals (dashed lines) or rested control animals (continuous line).

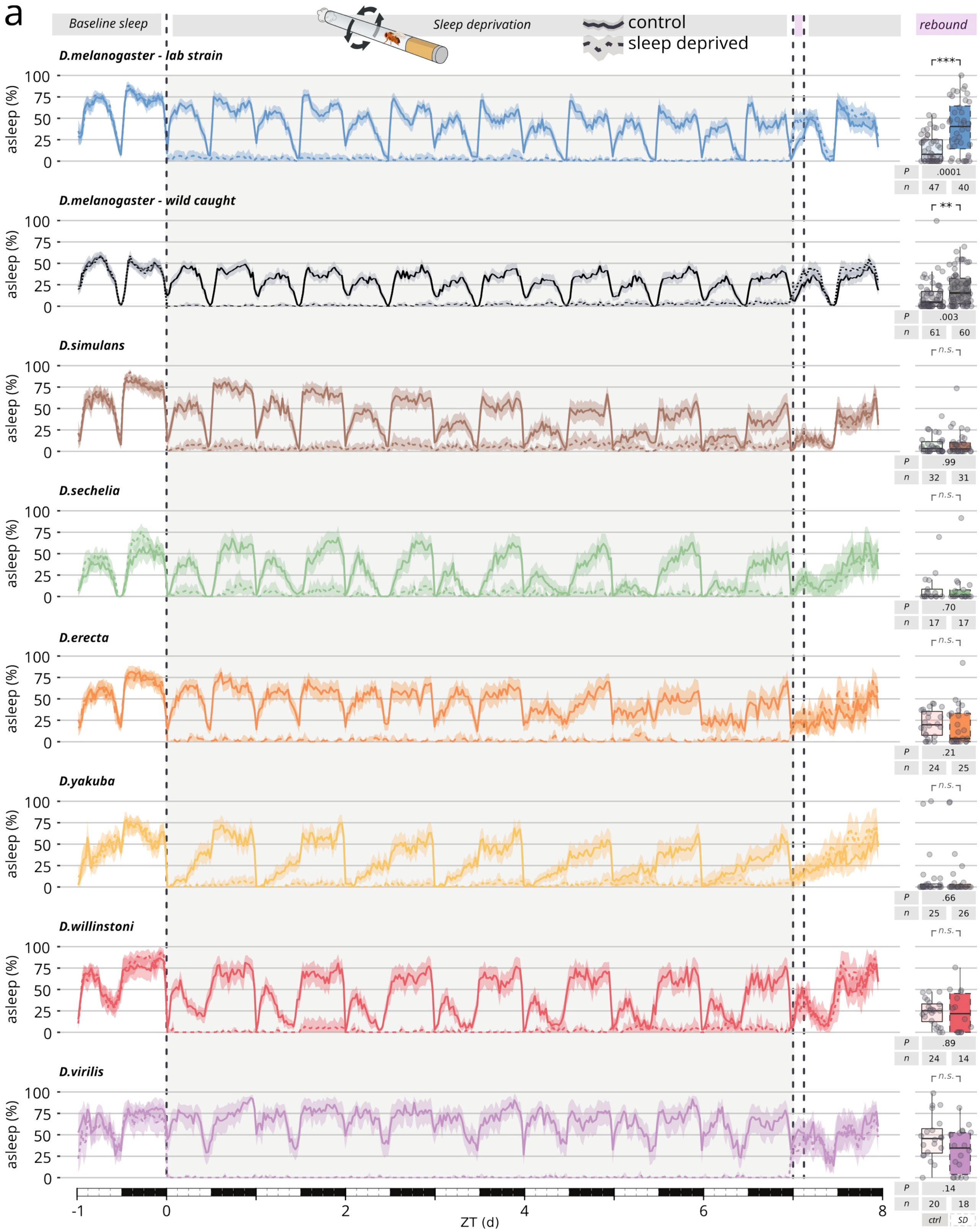

Supplementary Figure 4 | Chronic, seven days long sleep deprivation in the tested species.

a, Total sleep profile (left) and quantification of rebound (right) in flies subjected to seven continuous days of mechanically-induced sleep deprivation. Each panel features one strain from each of the seven wild-caught species, or CantonS. In all panels, the sleep profile of rested flies is shown as a continuous line, while sleep-deprived animals are shown in a dashed line. The sleep rebound at ZT 0-3 is quantified on the right side of each sleep profile. Numbers of animals (Ns) and P-values of sleep-deprived vs control are shown below each panel. *** P<0.001; ** P<0.01; * P<0.05.

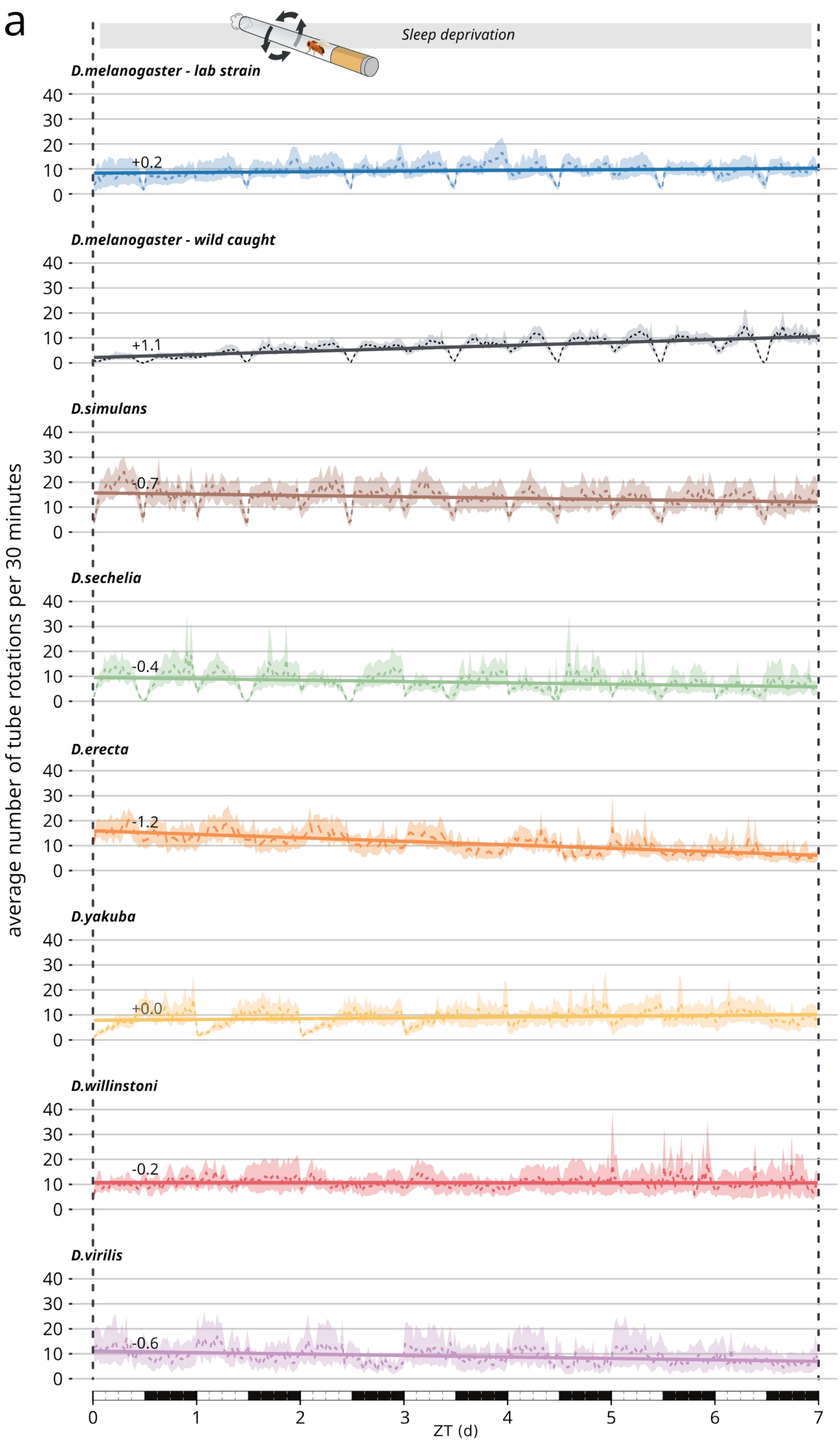

Supplementary Figure 5 | No signs of sleep pressure build-up during seven days long sleep deprivation in most species.

a, The cumulative average number of tube rotations in 30 minutes bins in all tested species (dashed lines) and the relative trend (continuous line) calculated as time series regression linear model. The numbers above each trend line indicate the coefficient. All regressions have P-values smaller than 10^{-6}, except for *D. Yakuba* that has a p-value of 0.99.

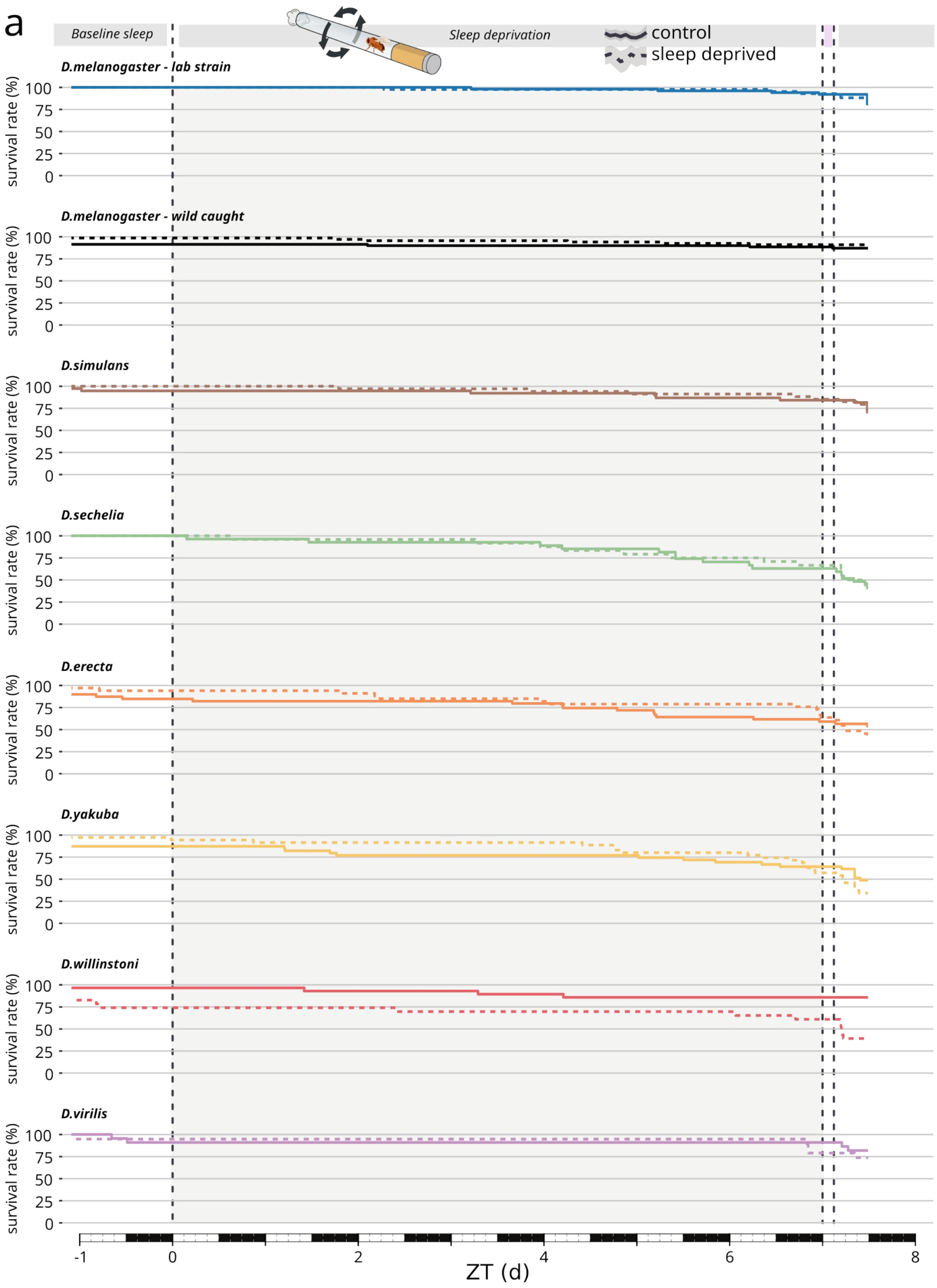

Supplementary Figure 6 | No lethality after seven days of sleep deprivation in any of the species.
a, Survival curves for flies subjected to chronic, seven days long, sleep deprivation, by species.

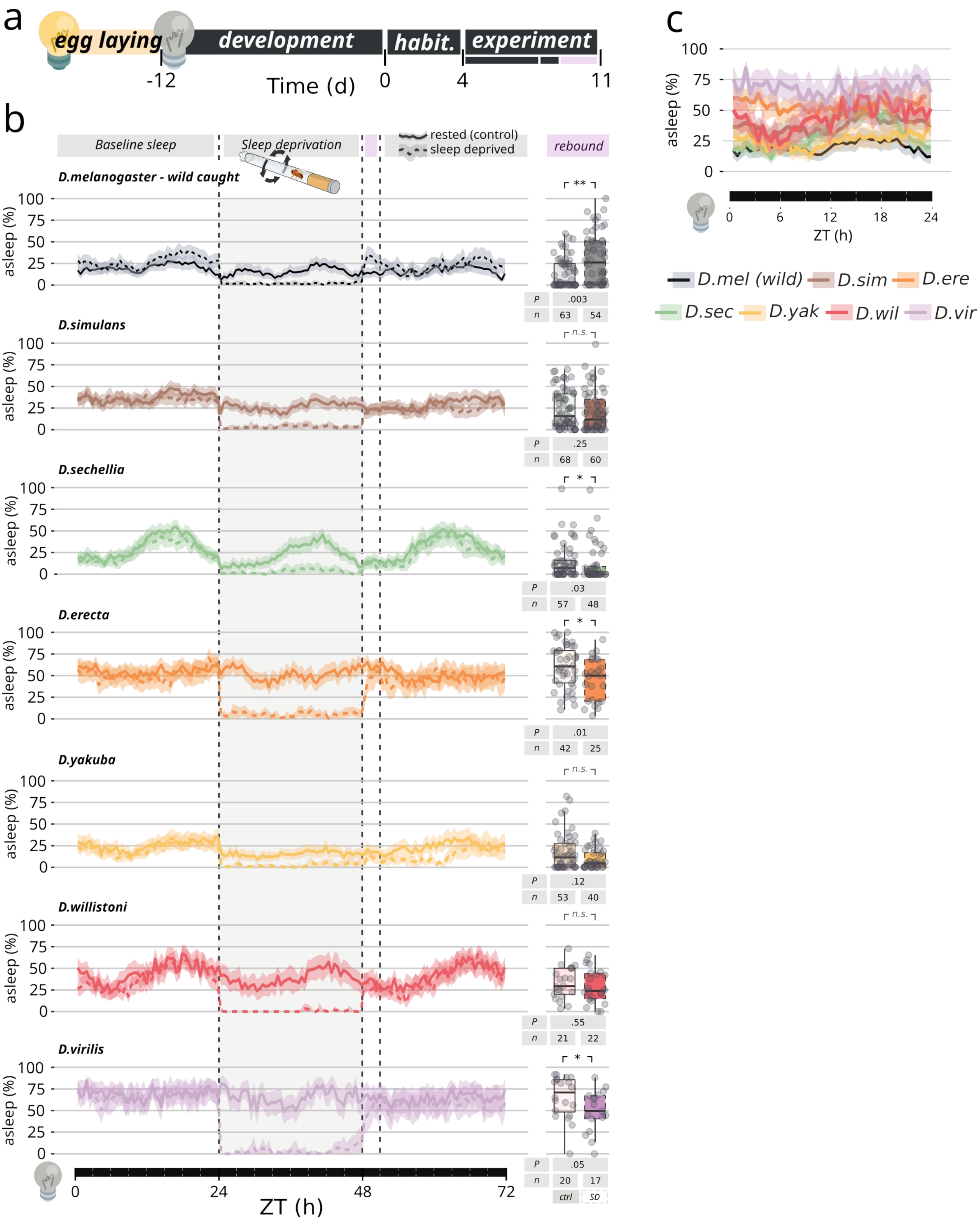

Supplementary Figure 7 | No sleep rebound, even in absence of external zeitgebers.
a, Schematic description of the experimental paradigm. Mated female flies were kept in constant light condition to attempt disruption of their circadian clock during egg laying. After laying, embryos were transferred to constant darkness and let develop, emerge, habituate without experiencing any light zeitgeber. At around 2-3 days of age, male flies were transferred to ethoscope tubes under conditions of faint red illumination. The time of transfer was randomly staggered throughout the day to avoid mechanical entrainment due to the experimenters' manipulation. Ethoscope analysis and mechanical sleep deprivation were also conducted in constant darkness. **b,** 72 h sleep profile (left) and quantification of rebound (right) in

flies subjected to 24 h of mechanically-induced sleep deprivation in constant darkness, treated as depicted in **a**. Each panel features one strain from each of the seven wild-caught species, or CantonS. In all panels, the sleep profile of rested flies is shown as a continuous line, while sleep-deprived animals are shown in a dashed line. The sleep rebound at ZT 0-3 is quantified on the right side of each sleep profile. Numbers of animals (Ns) and P-values of sleep-deprived vs control are shown below each panel. *** P<0.001; ** P<0.01; * P<0.05. **c**, Overlapping plot of baseline sleep in constant DD conditions for all seven species. Data are extracted from the dataset in b and highlight that some limited rhythmicity remains in some of the species (e.g. *D. willistoni* and *D. sechelia*) even when these were developed and raised in total darkness as mentioned in the methods (Re: Circadian analysis in constant darkness).

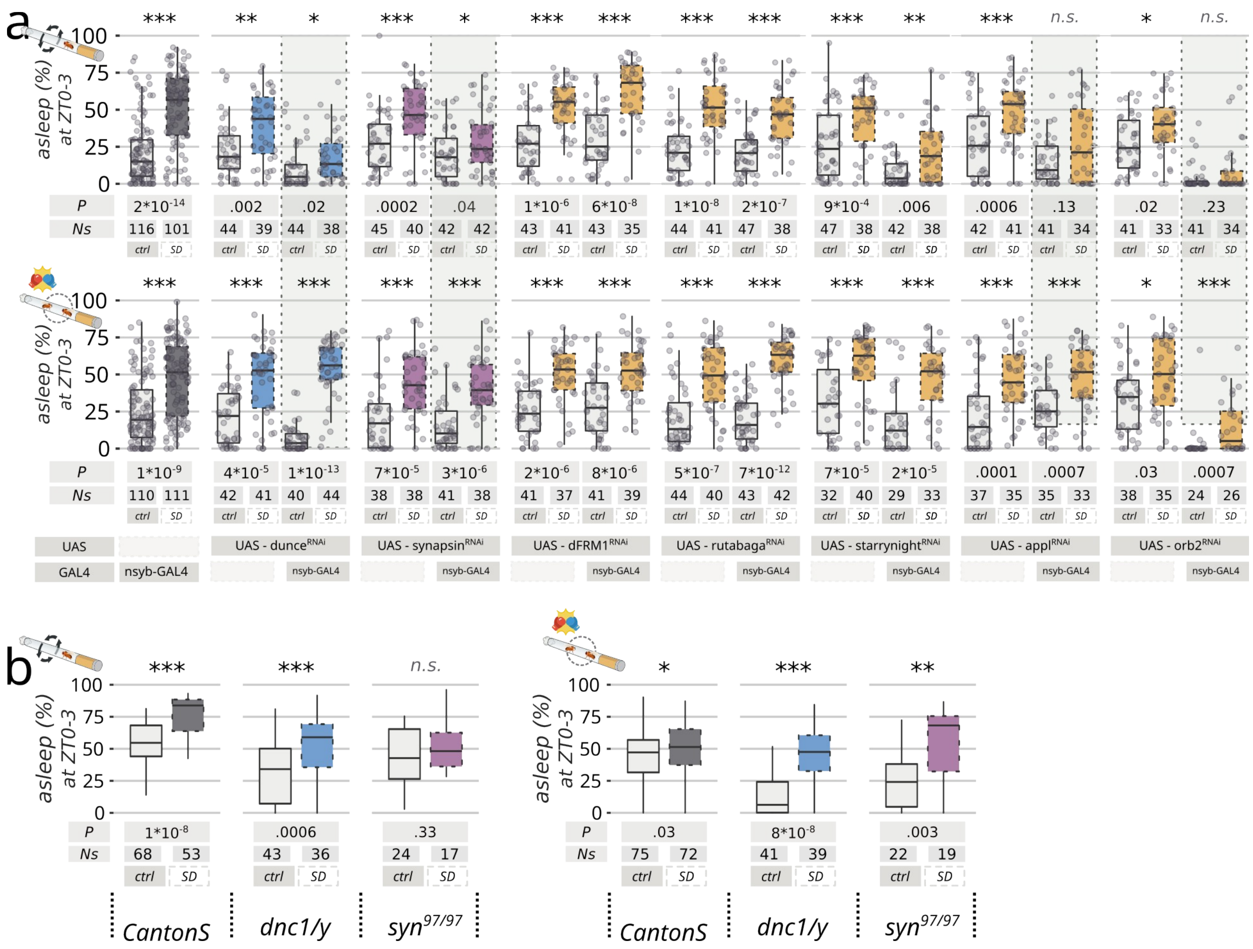

Supplementary Figure 8 | Detailed rebound for synaptic strength manipulations.

a, Rebound sleep at ZT0-3 for flies that underwent RNAi knock-down in one of seven selected genes using the pan-neuronal GAL4 driver nSyb and their relevant parental controls. Same dataset as Fig. 3A but different representation and statistical analysis. **b,** Rebound sleep at ZT0-3 after 24 h of mechanical (top) or male-male (bottom) sleep deprivation in dunce hemizygous mutants (blue) or synapsin homozygous mutants (purple) compared to wild-type CantonS flies.
