# Supplementary material for: Divergent evolution of sleep in Drosophila species
Source: Nat Commun. 2024 Jun 14;15:5091. doi: 10.1038/s41467-024-49501-9 (PMC11178934; doi:10.1038/s41467-024-49501-9)
Supplement: Supplementary file 3 — Reporting Summary [file 41467_2024_49501_MOESM3_ESM.pdf]

nature portfolio

Corresponding author(s): Giorgio F. GILESTRO

Last updated by author(s): Apr 15, 2024

# Reporting Summary

Nature Portfolio wishes to improve the reproducibility of the work that we publish. This form provides structure for consistency and transparency in reporting. For further information on Nature Portfolio policies, see our Editorial Policies and the Editorial Policy Checklist.

## Statistics

For all statistical analyses, confirm that the following items are present in the figure legend, table legend, main text, or Methods section.

| n/a | Confirmed | |
|---|---|---|
| ☐ | ☒ | The exact sample size (*n*) for each experimental group/condition, given as a discrete number and unit of measurement |
| ☐ | ☒ | A statement on whether measurements were taken from distinct samples or whether the same sample was measured repeatedly |
| ☐ | ☒ | The statistical test(s) used AND whether they are one- or two-sided<br>*Only common tests should be described solely by name; describe more complex techniques in the Methods section.* |
| ☐ | ☒ | A description of all covariates tested |
| ☐ | ☒ | A description of any assumptions or corrections, such as tests of normality and adjustment for multiple comparisons |
| ☐ | ☒ | A full description of the statistical parameters including central tendency (e.g. means) or other basic estimates (e.g. regression coefficient) AND variation (e.g. standard deviation) or associated estimates of uncertainty (e.g. confidence intervals) |
| ☐ | ☒ | For null hypothesis testing, the test statistic (e.g. *F*, *t*, *r*) with confidence intervals, effect sizes, degrees of freedom and *P* value noted<br>*Give P values as exact values whenever suitable.* |
| ☐ | ☒ | For Bayesian analysis, information on the choice of priors and Markov chain Monte Carlo settings |
| ☐ | ☒ | For hierarchical and complex designs, identification of the appropriate level for tests and full reporting of outcomes |
| ☒ | ☐ | Estimates of effect sizes (e.g. Cohen's *d*, Pearson's *r*), indicating how they were calculated |

*Our web collection on statistics for biologists contains articles on many of the points above.*

## Software and code

Policy information about availability of computer code

| | |
|---|---|
| Data collection | Behavioural data were collected using the open source ethoscope platform (Geissmann et al 2017 and https://lab.gilest.ro/ethoscope).<br>Images of immuno-stained brains were collected using a LEICA SP8 microscope with its proprietary acquisition software. |
| Data analysis | Behavioural data were analysed using the open source R package rethomics (Geissmann et al 2019, https://lab.gilest.ro/rethomics) or the open source Python package ethoscopy (Blackhurst et al 2023, https://lab.gilest.ro/ethoscopy). Jupyter notebooks of each analysis are provided as supplementary data in the openly accessible Zenodo repository with DOI 10.5281/zenodo.10554851<br>Software versions used to analyse the data were as follows: behavr: 0.3.2; sleepr: 0.3.0; zeitgebr: 0.3.3; ggetho: 0.3.4; scopr: 0.3.3; ethoscopy 1.3.5. |

For manuscripts utilizing custom algorithms or software that are central to the research but not yet described in published literature, software must be made available to editors and reviewers. We strongly encourage code deposition in a community repository (e.g. GitHub). See the Nature Portfolio guidelines for submitting code & software for further information.

## Data

Policy information about availability of data

All manuscripts must include a data availability statement. This statement should provide the following information, where applicable:

- Accession codes, unique identifiers, or web links for publicly available datasets
- A description of any restrictions on data availability
- For clinical datasets or third party data, please ensure that the statement adheres to our policy

All the raw and processed data are made available freely through the Zenodo repository with DOI 10.5281/zenodo.10554851

## Research involving human participants, their data, or biological material

Policy information about studies with human participants or human data. See also policy information about sex, gender (identity/presentation), and sexual orientation and race, ethnicity and racism.

| | |
|---|---|
| Reporting on sex and gender | N/A |
| Reporting on race, ethnicity, or other socially relevant groupings | N/A |
| Population characteristics | N/A |
| Recruitment | N/A |
| Ethics oversight | N/A |

Note that full information on the approval of the study protocol must also be provided in the manuscript.

# Field-specific reporting

Please select the one below that is the best fit for your research. If you are not sure, read the appropriate sections before making your selection.

☒ Life sciences ☐ Behavioural & social sciences ☐ Ecological, evolutionary & environmental sciences

For a reference copy of the document with all sections, see nature.com/documents/nr-reporting-summary-flat.pdf

# Life sciences study design

All studies must disclose on these points even when the disclosure is negative.

| | |
|---|---|
| Sample size | The determination of the sample size for this study was guided by prior experience, indicating that a minimum of 30-40 animals per group is required to ensure robust and reliable results. |
| Data exclusions | Some data points had to be excluded a priori for the analysis, mostly due to hardware failures in the sleep deprivation module. A record of those events is kept in the metadata files which are publicly shared along with the raw data and the scripts in the Zenodo repository. |
| Replication | All results were reproduced and at least two independent replicates were performed for each experiment. The metadata tables provide information on the dates and condition for each experiment. |
| Randomization | When performing behavioural analyses, flies were spread out randomly through the machines to avoid flies of the same genotype/condition to all be analysed by the same machine. Immunohistochemistry was performed on randomised animals and score blindly (see below). |
| Blinding | Behavioural experiments were not blinded to condition/genotype during the animal manipulation but data collection and analysis was done in a non-biased manner using only automatic analysis through batch scripts and automatic data fetching from the machines. Immunohistochemistry was scored blindly. |

# Reporting for specific materials, systems and methods

We require information from authors about some types of materials, experimental systems and methods used in many studies. Here, indicate whether each material, system or method listed is relevant to your study. If you are not sure if a list item applies to your research, read the appropriate section before selecting a response.

## Materials & experimental systems

| n/a | Involved in the study | |
|---|---|---|
| ☐ | ☒ | Antibodies |
| ☒ | ☐ | Eukaryotic cell lines |
| ☒ | ☐ | Palaeontology and archaeology |
| ☐ | ☒ | Animals and other organisms |
| ☒ | ☐ | Clinical data |
| ☒ | ☐ | Dual use research of concern |
| ☒ | ☐ | Plants |

## Methods

| n/a | Involved in the study | |
|---|---|---|
| ☒ | ☐ | ChIP-seq |
| ☒ | ☐ | Flow cytometry |
| ☒ | ☐ | MRI-based neuroimaging |

# Antibodies

Antibodies used

Primary: anti-nc82 (Ab 2314866 DSHB)
Secondary: #1 AB150115 Goat Anti-Mouse IgG H&L (Alexa Fluor® 647)

Validation

The anti-nc82 is a commercially available antibody validated extensively in the literature. First used for this scope in Gilestro et al 2009. No other antibody was used in this study.

# Animals and other research organisms

Policy information about studies involving animals; ARRIVE guidelines recommended for reporting animal research, and Sex and Gender in Research

Laboratory animals

The following VDRC-RNAi transgenic strains were used in this study: UAS-dunceRNAi (#107967), UAS-synapsinRNAi (#109587), UAS-dFRM1RNAi (#110800), UAS-rutabagaRNAi (#101759), UAS-starrynightRNAi (#107993), UAS-applRNAi (#108312) and UAS-orb2RNAi (#11753). The nSyb-GAL4 and MB010B-GAL4 were gifted to us by Crystal Vincent (Imperial College London, UK) and Andrew Lin (The University of Sheffield, UK) respectively, while the other GAL4 lines were obtained from Bloomington Drosophila Stock Centre (BDSC, Indiana, USA): R30G03-GAL4 (#49646), R52B10-GAL4 (#69657), R58E02-GAL4 (#41347), R72G06-GAL4 (#39792). All RNAi and GAL4 lines were outcrossed for six generations to the w1118 background before testing. The dunce1 (#6020), synapsin97 (#29031), OregonR (#5) and w1118 (#3605) flies, were also obtained from BDSC; CantonS strain originally from Ralf Stanewsky (Münster University, Germany). The two D. melanogaster wild-caught strains were a gift of Darren Obbard (University of Edinburgh, UK). Two strains of non-melanogaster species were used in Fig. 1C, and the first strain of each were studied in the succeeding figures: D.simulans (14021-0251.254 #60, 14021-0251.196 #61), D.sechellia (14021-0248.25 #3, 14021-0248.28 #53), D.erecta (14021-0224.01 #11), D.yakuba (14021-0261.01 #5, 14021-0261.48 #51), D.willistoni (14030-0811.24 #1, 14030-0811.13 #55) and D.virilis (15010-1051.87 #9, 15010-1051.118 #54). The wildtype species and the white-eyed "intruder" species: D.simulans w- (14021-0251.133), D.sechellia w- (14021-02048.30), D.erecta w- (14021-0248.30), D.yakuba w- (14021-0261.04), D.willistoni w- (14030-10811-33) and D.virilis w- (15010-1051.45) were acquired from The National Drosophila Species Stock Center (NDSSC, Cornell University, USA). All experiments were started when were 6-10 days of age.

Wild animals

No wild animals were collected directly in this study. The species defined as wild were collected in previous work by Darren Obbard ( https://doi.org/10.1371/journal.ppat.1007050 )

Reporting on sex

Sex of the animals used was always reported in the figure legends or methods section.

Field-collected samples

No samples were field collected.

Ethics oversight

Not necessary.

Note that full information on the approval of the study protocol must also be provided in the manuscript.

# Plants

Seed stocks

N/A

Novel plant genotypes

N/A

Authentication

N/A
